# The aprotinin saga and the risks of conducting meta-analyses on small randomised controlled trials – a critique of a Cochrane review

**DOI:** 10.1186/1472-6963-9-34

**Published:** 2009-02-19

**Authors:** Måns Rosén

**Affiliations:** 1 SBU (The Swedish Council on Technology Assessment in Health Care), Box 5650, SE-114 86 Stockholm, Sweden

## Abstract

**Background:**

Aprotinin for reducing blood loss during coronary artery bypass surgery was withdrawn from the market after early termination of a large randomised controlled trial (RCT) showing excess mortality for patients receiving aprotinin compared to lysine analogues. Several meta-analyses of small RCTs did not show excess mortality and even indicated reduced mortality, while several observational studies showed excess mortality. The aim of this paper is to review the quality of the meta-analysis of a Cochrane report.

**Methods:**

The 52 studies included in the meta-analysis of the Cochrane report were reviewed according to whether an objective to study mortality was formulated in advance, whether follow-up method or time were specified, and whether the study had statistical power to show any effect.

**Results:**

The Cochrane report restricted the analysis to RCTs, but the largest study should not have been included given that it was a prospective observational study with 1 784 patients rather than an RCT. None of the RCTs had sufficient statistical power to detect differences in mortality. Most studies had fewer than 100 patients. Seven out of 51 RCTs had mortality outcome as one of their objectives. Only very few described follow-up method or time.

**Conclusion:**

It is doubtful whether small studies should be included in meta-analyses if they do not have the purpose of studying the specified outcome and if the follow-up method or time are not adequately described. The aprotinin saga shows overconfidence in small RCTs of inferior quality compared to well-conducted observational studies.

## Background

Aprotinin to reduce blood loss during coronary artery bypass surgery was approved by the U.S. Food and Drug Administration (FDA) in 1993 and has been a very common procedure since then. The BART study, which was recently published in the *New England Journal of Medicine *[1], showed that aprotinin for reducing blood loss during coronary artery bypass surgery increased mortality by 50 percent (RR = 1.53; 95% CI, 1.06–2.22) compared with groups receiving lysine analogues. Before BART, several meta-analyses of randomised controlled trials (RCTs) had shown no indication of an excess risk of death or had even shown a reduced risk [2-4], while several observational studies had shown excess mortality risks and increased risk of renal dysfunction [5-9]. The reasons for these discrepancies deserve further analysis.

The focus of the meta-analyses was usually aprotinin's ability to reduce blood loss during surgery. Aprotinin has been a successful treatment in this connection. Based on a cumulative meta-analysis of 64 trials, an editorial in *The Lancet *even claimed that many of the later studies were unnecessary since meta-analysis showed that the effects had already been established after the twelfth trial in 1992 [10]. This was probably true with respect to blood loss, but not with respect to mortality and other adverse effects. Blood loss is also primarily a surrogate endpoint.

The findings of the observational studies by Mangano [6,7], Schneeweiss [8] and Shaw [9] were based on prospective cohorts of 4 374, 78 199 and 10 275 patients respectively. They all showed statistically significant mortality odds ratios. The study by Mangano [6], which had an odds ratio of 1.48 (95% CI, 1.19–1.85), controlled for background characteristics such as age, gender, socioeconomic status, geographic region and medical history. The study by Schneeweiss [8] showed an odds ratio of 1.64 (95% CI, 1.50–1.78) adjusted for 41 background characteristics. Both of these studies showed a dose-response relationship, with higher mortality for higher doses of aprotinin. A case-control study by Karkouti et al used propensity scores and did not find any differences in adverse outcomes except for renal dysfunction, which was significantly higher in the aprotinin group [5].

The observational studies of aprotinin, which were much larger than the RCTs, controlled for many important background characteristics of the patients. The critics of these observational studies emphasised the limitations of observational studies in general, as well as uncertainty about whether all confounders had been controlled for. Selection bias may also have been present due to the fact that aprotinin was a preferred choice for patients with high risk of bleeding. These factors were probably among the main reasons that FDA did not take more forceful action between 2006 and late 2007.

One of the main purposes of meta-analysis is to summarise data from small studies to obtain more robust estimates of effects. However, this is appropriate only if the small RCTs are well-designed and well-conducted. In order to analyse whether this was the case, this author examined all the studies in one of the meta-analyses according to certain quality criteria. The meta-analysis chosen was a 2007 Cochrane report that reviewed anti-fibrinolytic use for minimising perioperative allogenic blood transfusion [2]. The report included assessments of both the benefits and the risks of aprotinin versus placebo or other treatment options. The review restricted the analysis to RCTs and referred to observational studies in the discussion section only.

Mortality was assessed in the Cochrane report by performing a meta-analysis of 52 small RCTs. The meta-analysis showed no excess risk for aprotinin versus placebo (RR = 0.90, 95% CI, 0.67–1.20 with a total of 7 721 patients and 192 deaths).

## Methods

All 52 studies were analysed in full text. The three questions raised were: Was mortality stated as an objective? Did the study have power to analyse adverse mortality effects? Was the method for follow-up clearly described? If mortality was stated as an objective and if statistical power was considered, it is an indication that the investigators intended to examine mortality thoroughly. A description of the follow-up method is very important if the reader is to have confidence in the results.

## Results

The 52 studies were assessed by analysing the formulated objectives, the specified follow-up method and time, and potential power to detect differences in mortality (Table 1). The largest study should not have been included given that it was a prospective observational study with 1 784 patients rather than an RCT. Most studies had fewer than 100 patients. Seven out of 51 (13.7%) RCTs had mortality outcome as one of the objectives. None of the studies had sufficient statistical power to detect differences in mortality. Only very few described the follow-up method. Follow-up time was specified in 16 out of 51 (31.4%) studies and varied from 24 hours, during hospitalisation, to 30 days to 3 months. Most studies did not show whether mortality was measured during surgery, hospital stay or longer follow-up periods.

**Table 1:** Characteristics of included RCT studies in the Cochrane meta-analysis of adverse mortality effects Aprotinin versus placebo.

**Reference, author**	**Adverse-mortality effects formulated as an outcome in the objective**	**Included patients [N] and design or power to analyse adverse mortality effects** **N**		**Method and follow-up period for studying adverse mortality effects**
Alvarez, 1995	No	100	No	Not specified

Bidstrup, 1989	No	80	No	Not specified

Bidstrup, 1993	No	90	No	Not specified, 7–12 days

Blauhut, 1994	No	43	No	Not specified

Casas, 1995	No	149	No	Not specified

Cosgrove, 1992	No	169	No	Not specified

D'Ambra, 1996	Yes	212	No	Yes, hospitalisation

Dietrich, 1992	Yes, but no RCT	1784	Yes	Yes, hospitalization

Dietrich, 1995	No	30	No	Not stated, at least 41 days

Ehrlich, 1998	No	50	No	Not stated, at least 30 days

Garcia-Huete, 1997	No	80	No	Not stated, at least 30 days

Green, 1995	Yes	84	No	Yes, 3 months after discharge.

Hardy, 1993	No	41	No	Not specified

Hayashida, 1997	Yes	167	No	Not stated, at least 12 days

Jamieson, 1997	No	91	No	Not specified

Lab, 1995	No	110	No	Yes, hospitalisation

Lemmer, 1996	Yes	704	No	Not stated

Lemmer, 1994	Yes	216	No	Yes, 30 days mortality

Levy, 1995	Yes	287	No	Not specified

Liu, 1993	No	40	No	Not specified

Maccario, 1994 [It]	No	99	No	Not stated

Mohr, 1992	No	50	No	Not specified

Okita, 1996	No	112	No	Not specified

Rocha, 1994	No	109	No	Yes, first 10 days

Royston, 1987	No	22	No	Not specified

Stammers, 1997	No	20	No	Yes, hospitalisation

Swart, 1994	Yes	100	No	Yes, 7 days after surgery

Ashrat, 1997	No	38	No	Not stated

Alderman, 1998	No	870	No	Yes, hospitalisation

Bidstrup, 2000	No	60	No	Not specified, 15 days reported

Cohen, 1998	Yes, maybe	115	No	Not specified

Misfeld, 1998	No	42	No	Not specified, hospitalisation

Nuttall, 2000	No	168	No	Not specified, 24 hours

Schweizer, 2000	No	57	No	Not specified

Moran, 2000	No	42	No	Yes, 3 months

Murkin, 2000	No	298	No	Yes, 3 months

Alvarez, 2001	No	55	No	Not specified

Dignan, 2001	No	202	No	Not specified

Findlay, 2001	No	63	No	Yes, hospitalisation

Kyriss, 2001	No	38	No	Not specified

Golanski, 2000	No	54	No	Not specified

Palmer, 2003	No	100	No	Yes, 3 months

Englberger, 2002a	No	29	No	Yes, hospitalisation

Englberger, 2002b	No	47	No	Yes, hospitalisation

Kipfer, 2003	No	30	No	Not specified

Rodrigus, 1996	No	93	No	Not specified

Kuepper, 2003	No	120	No	Yes, 24 hours after surgery

Van der Linden, 2005	No	75	No	Probably 30 days

Kunt, 2005	No	86	No	Probably 1 day, operative deaths

Koster, 2004	No	200	No	Yes, during hospitalisation

Diprose, 2005	No	198	No	Not stated

Kuitunen, 2005	No	60	No	Not stated

Footnote: The complete references can be found in the Cochrane review by Henry DA et al.[2].

## Discussion

The analysis was restricted to RCTs, but the largest of the 52 included studies was an observational study by Dietrich et al [11] and was therefore erroneously included in the meta-analysis. This study was conducted was conducted before the FDA approval in 1993 when there was no suspicion of adverse effects. This is apparent from the study design with no focus on mortality and no control for confounding factors. Analysis of all the included RCTs in the Cochrane review of mortality showed that a minority of the studies had mortality outcome as one of their objectives. Nor did any of the studies have statistical power to detect differences in mortality. This clearly indicates the investigators' lack of focus on mortality and adverse events. However, this does not mean *a priori *that the studies lacked quality, although it does indicate that less time was spent on this part of the study design.

The fact that most studies completely lacked a description and specification of follow-up method or time should raise more serious concerns. Many studies did not specify whether deaths occurred during surgery, during hospital stay or within a specified period of time. It is questionable whether it is appropriate to perform a meta-analysis with very different follow-up times or when most of the studies did not specify follow-up time at all. Mortality is not an adverse effect that occurs during hospitalisation only. Severe complications may lead to death long after discharge from hospital. For example, thrombotic events can manifest clinically over a period of months or years following arterial occlusion [12,13]. In order to properly analyse mortality, follow-up time must be specified.

The results of this paper could contribute to the ongoing debate concerning the value of large versus small trials. Previous studies have suggested that publication bias is one reason for discrepancies between the results of large RCTs and meta-analyses of small RCTs [14-16]. Kjaergard et al [17] focused on methodological quality and concluded that inadequate generation of the allocation sequence, allocation concealment and double blinding lead to exaggerated estimates of benefits and may contribute to discrepancies between the results of large RCTs and meta-analyses of small RCTs. In general, it seems likely that large studies requiring more resources and funds have undergone a more thorough review process than small studies. The results of the present paper also indicate the need to analyse the quality of studies before including them in a meta-analysis. Studies cannot be included if the method of following up outcome has not been specified. Already before the publication of the BART study, an editorial in the *New England Journal of Medicine *discussed the limitations of these small trials and argued that they should have been interpreted more cautiously, particularly given the contradictory findings of some epidemiological studies [18].

Another issue raised by the aprotinin saga is the value of observational studies in examining rare and adverse outcomes such as mortality. The advantages of large, well-conducted RCTs are undisputable. But even large RCTs rarely have the ability to show significant results concerning rare outcomes and usually have short follow-up periods. In the case of aprotinin, data from several well-conducted and very large observational studies were available between 2006 and late 2007 without any major action being taken. Two of the studies also showed a dose-response relationship, with higher mortality odds ratios for higher doses of aprotinin [7,8] A 2006 case-control study and a 2007 meta-analysis of RCTs showed that high-dose aprotinin significantly increased the risk of renal dysfunction [19]. The main reasons for not taking action seem to be confidence in small RCTs that did not show any adverse effects and mistrust of observational studies that showed excess mortality. In this case, the consistent findings of the observational studies should have been more carefully considered, especially when other measures to reduce bleeding were available. In retrospect, it seems clear that aprotinin would have been withdrawn from the market by the company earlier if FDA and others had taken well-conducted observational studies more seriously.

## Conclusion

The RCTs included in the Cochrane review had blood loss as the main outcome and were not suitable for studying mortality. Methods were not well-specified and had serious limitations. In such cases, meta-analysis is a questionable approach, considering the methodological limitations of such small trials.

## Competing interests

The author declares that they have no competing interests.

## Response

Response by David Henry

Email: david.henry@newcastle.edu.au

Address: University of Newcastle, Faculty of Health, Level 5, Clinical Sciences Building, Newcastle Mater Hospital, Waratah, NSW, Australia, 2298.

Professor Måns Rosén raises concerns about our 2007 Cochrane review of anti-fibrinolytic drugs [2]. The topic is an important one – many patients receive these drugs as an adjunct to reduce blood loss during a variety of surgical procedures. Some of his concerns go to the heart of assumptions that are made when conducting meta-analyses of randomized controlled clinical trials.

First, we should point out that we have updated some of the meta-analyses of trials contained in the Cochrane review and have recently published the results. This analysis is confined to the use of anti-fibrinolytic trials in cardiac surgery. The full text is available at [20]. In the updated report we found an increased risk of death in subjects treated with aprotinin compared with tranexamic acid or aminocaproic acid. Our conclusions were "The risk of death tended to be consistently higher with use of aprotinin than with use of lysine analogues. Aprotinin had no clear advantages to offset these harms...The conclusions of our updated review conflict with those of our published Cochrane review.... The addition of data from the large BART study increased the relative risk of death with the use of aprotinin compared with the use of either tranexamic acid or epsilon aminocaproic acid."

So, the updated review is consistent with the observational studies of aprotinin (recently presented in a systematic review [21]) in finding an increased risk of death with aprotinin compared with the lysine anti-fibrinolytic agents. But significantly, the updated meta-analysis found no increase in the risk of death with aprotinin compared with placebo/no treatment when used in cardiac surgery (summary RR for death 0.93; 95% CI 0.69, 1.25). This summary analysis comprised results from forty-nine trials of aprotinin, which included 7439 participants and reported on 182 deaths. This finding troubles Professor Rosén as it is at odds with the results of large observational studies and is based on small randomized trials that were not designed to show a change in the incidence of death or indeed cardiovascular events. It should be noted here that neither was the BART study.

The five main criticisms made by Rosén are: that few of the trials had mortality as a stated outcome; none of the trials had statistical power to detect differences in mortality; very few trials described the follow-up methods and follow-up time was not specified in most of them; follow-up may have been too short to quantify late deaths. One large study was not a randomized trial. While we don't dispute the accuracy of these observations about the individual studies we disagree with the inferences that Professor Rosén makes regarding the systematic review.

If at least one large trial had specified thrombosis (a theoretical adverse effect of these drugs) and death as outcomes, and had adequate power and sufficient follow-up, there would have been little need for the meta-analysis. One of the purposes of systematic reviews is to examine events that were not primary outcomes of the individual trials. By denying this Rosén is arguing against a central purpose of systematic reviews. In terms of patient follow-up, this varied between the aprotinin trials, but should not have varied between the treatment and control arms of the individual trials. In other words, it is unlikely that this was a source of bias either toward or away from the null. While some non-fatal events could lead to late mortality, we found no increase in the risk of non-fatal thrombosis. In addition, the BART trial3, cited by Rosén, found a separation of survival curves early in the post-operative phase and the curves are roughly parallel from Day 10 onwards. So, it is unlikely that undetected late mortality accounts for the lack of risk that we found in the meta-analysis of the placebo/inactive controlled aprotinin trials.

However, we do share one of Rosén's worries, which have also been expressed by Ray [22]. This concerns the completeness of reporting of uncommon events in small clinical trials. We put considerable effort into identifying trials that appeared to report mortality, but we have no way of assessing how rigorously this was done. One concern about trials of drugs is the tradition to report 'adverse reactions' – events that are reported as 'possibly' or 'probably' caused by the drug. The use of causality assessment, traditional in the assessment of voluntary adverse reaction reports, could lead to under-reporting of events. This process has no place in the reporting of the results of clinical trials.

Regarding the alleged inclusion of one non-randomized study in the meta-analysis, the methods section in this study states: "Patients were randomly assigned to either an aprotinin treatment group (group A) or to a control group without aprotinin (group C)". We accepted this information in good faith, but scored the methodological quality of the study as low. We did not routinely contact study authors to confirm the details of randomization. Like most meta-analysts we accepted and scored the written description of the methods. We subsequently contacted the senior author, Professor Wulf Dietrich of the University of Munich, who kindly reviewed his files (the study was reported in 1992 [11]) and has advised us that in his opinion the study does not meet contemporary standards for being considered 'randomized', that there was a possibility of selection bias, but this would have led to sicker patients receiving aprotinin. It should be noted that exclusion of this study does not change the overall estimate of mortality with aprotinin compared with control: Pooled RR = 1.02 (95%CI 0.71 to 1.47).

So did our Cochrane review miss an adverse effect of aprotinin? That is possible. Are there significant problems with meta-analyses of infrequent outcomes measured in small clinical trials? Yes there are. But in our view the major criticisms voiced by Professor Rosén concerning the specification of outcomes, statistical power of individual studies and variable follow up of trial participants are not the key issues. Under-reporting of infrequent events is pivotal and if non-differential (the most likely scenario) will lead to a bias to the null. This could account for the fact that we found no increase in mortality in the aprotinin trials. We have acknowledged this in the updated review.3 Despite considerable methodological improvements, meta-analysis remains an imperfect science, being an observational not an experimental discipline, which relies heavily on the diligence of trial investigators and authors of reports.

Systematic reviews must be rigorously performed, but Professor Rosén has not made a comprehensive assessment of the quality of our work. Tools exist to enable appraisal of systematic reviews (for instance the recently validated AMSTAR instrument) [23]. Systematic reviews have value in summarizing literature, providing overall estimates of effect and in assisting in the planning of clinical trials. In the latter regard it is significant that two of the authors of the Cochrane review (Fergusson and Laupacis [1]) were involved in the planning conduct and monitoring of the BART trial. This trial owed a lot to the results of the many published meta-analyses of this literature.

## Pre-publication history

The pre-publication history for this paper can be accessed here:


